# Cost-Effectiveness Analysis of Innovative Therapies for Patients with Non-Alcoholic Fatty Liver Disease

**DOI:** 10.3390/jmahp12020005

**Published:** 2024-04-02

**Authors:** Michal Pochopien, Jakub Wladyslaw Dziedzic, Samuel Aballea, Emilie Clay, Iwona Zerda, Mondher Toumi, Borislav Borissov

**Affiliations:** 1 Assignity, Wadowicka 8a, 30-415 Krakow, Poland; 2 InovIntell, 3023GJ Rotterdam, Zuid-Holland, The Netherlands; 3 Clever-Access, 53 Avenue Montaigne, 75008 Paris, France; 4 InovIntell, 215 rue du Faubourg St Honoré, 75008 Paris, France; 5 Prescriptia, 28, Hristo Botev Blvd., 1517 Sofia, Bulgaria

**Keywords:** NASH, NAFL, Markov cohort model, economic evaluation, cost-effectiveness, nonalcoholic fatty liver, non-alcoholic steatohepatitis

## Abstract

Objective: Currently there are no disease-specific approved therapies for non-alcoholic fatty liver (NAFL) and non-alcoholic steatohepatitis (NASH); however, several treatments are under development. This study aimed to estimate the cost-effectiveness of hypothetical innovative therapies compared with lifestyle intervention alone and combined with pioglitazone, and assess the health economic consequences of their future availability for patients. Methods: A Markov cohort model was developed, considering fourteen disease health states and one absorbing state representing death. Transition probabilities, costs, utilities, and treatment efficacy were based on published data and assumptions. Four treatment strategies were considered, including two existing therapies (lifestyle intervention, small molecule treatment) and two hypothetical interventions (biological and curative therapy). The analysis was performed from the US third-party payer perspective. Results: The curative treatment with the assumed efficacy of 70% of patients cured and assumed price of $500,000 was the only cost-effective option. Although it incurred higher costs (a difference of $188,771 vs. lifestyle intervention and $197,702 vs. small molecule), it generated more QALYs (a difference of 1.58 and 1.38 QALYs, respectively), resulting in an ICER below the willingness-to-pay threshold of $150,000 per QALY. The sensitivity analyses showed that the results were robust to variations in model parameters. Conclusions: This study highlighted the potential benefits of therapies aimed at curing a disease rather than stopping its progression. Nonetheless, each of the analyzed therapies could be cost-effective compared with lifestyle intervention at a relatively high price.

## 1. Introduction

**Figure 1:**
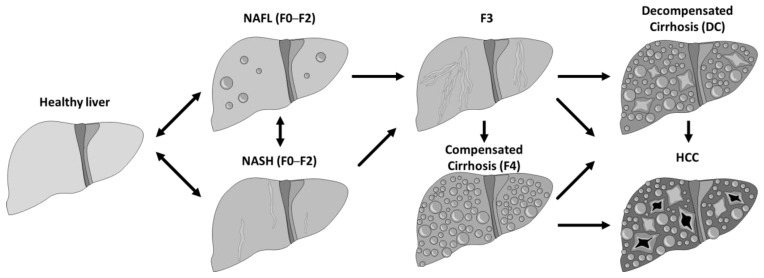
Natural progression of the NASH/NAFL disease.

Non-alcoholic fatty liver disease (NAFLD) comprises a spectrum of hepatic conditions closely associated with metabolic syndrome, including non-alcoholic fatty liver (NAFL) or hepatic steatosis, and non-alcoholic steatohepatitis (NASH) or steatohepatitis [1]. Patients classified with NAFLD can progress or regress between different fibrosis stages (F0 to F2) of NASH or NAFL and vice versa. NAFL generally follows a benign non-progressive clinical course, and NASH may progress to cirrhosis (F4), decompensated cirrhosis (DC), and hepatocellular carcinoma (HCC) (Figure 1).

NAFLD poses a major public health concern with a rising prevalence, becoming a leading cause of worldwide liver disease [2]. The global prevalence of NAFLD is estimated to be 30.05% among the adult population, with the highest prevalence in Latin America (44.37%), followed by Middle East and North Africa (MENA) (36.53%) [3]. The estimated prevalence of NASH among biopsied NAFLD patients is 59.10% worldwide [3]. This indicates that the overall prevalence of NASH ranges between 1.50% and 6.45% worldwide [4,5]. NASH is the second leading indicator of chronic liver diseases for liver transplantation (LT) in the United States following alcohol-related disease (38%), accounting for 28% of patients. In Europe, it represents 8.4% of annual LTs [4,5]. Metabolic comorbidities associated with NAFL and NASH include obesity (51.34%), type 2 diabetes (22.51%), hyperlipidemia (69.16%), hypertension (39.34%), and metabolic syndrome (42.54%), based on the provided global statistical review [3]. The liver-specific and overall global mortality rates in patients with NASH were 11.77 and 25.56 per 1000 person-years, respectively, in 2016 [3].

There are currently no disease-specific approved therapies for NAFL or NASH, however there are constantly new developments regarding the identification of this disease [6]. Lifestyle intervention remains the standard of care, with limited evidence of reducing liver fibrosis. In contrast, pioglitazone or vitamin E, used as add-on therapy to the standard of care, have shown potential in reversing steatohepatitis and improving liver fibrosis [7]. The pathophysiology of NASH is complicated and poorly understood, which has hindered progress in finding effective treatments. Given the multifactorial nature of the disease, there is ongoing interest in exploring combination therapies and diverse modes of action. In May 2019, the first series of studies focusing on cell-based therapy was launched to evaluate safety in NASH F3 and F4 patients at different dosages [8], building upon interim results from a phase I/II trial involving 19 patients with acute liver failure [9]. Over the course of nearly three years, studies on cell-based therapies and liver treatments have significantly increased. The summary of currently ongoing trials holds promise for the development of more efficient treatments [10,11].

Given the large number of patients with NAFL and NASH worldwide, medical costs related to these conditions are enormous, in both affluent and developing countries [12]. The financing of potential treatments for NAFL and NASH currently represents an area of uncertainty, considering the increasing number of pipeline agents advancing to late-phase clinical trials. In addition to demonstrating clinical efficacy, the evidence of long-term cost-effectiveness in a budget-constrained world is becoming increasingly critical. Previous attempts have been made to build models assessing the cost-effectiveness of treatments for NAFL and NASH [13,14,15]. However, these models did not incorporate the detailed interplay between NAFL and NASH, which can significantly influence the results. Furthermore, there was no differentiation across various fibrosis stages in the reviewed models. 

The objective of this study was to estimate the cost-effectiveness of hypothetical innovative therapies compared with lifestyle intervention alone, as well as in combination with a small molecule (pioglitazone). Additionally, the study aimed to identify the key factors that drive cost-effectiveness and investigate economically justifiable prices. Therefore, this paper aims to focus on developing a more comprehensive model without becoming overly complex.

In our investigations, we wanted to understand the potential of different therapies for NAFL and NASH from the perspective of their economic evaluation. Thus, we used lifestyle intervention as a baseline and compared it with three other treatments (small molecule, biological, and curative). Each intervention had an assumed mode of action and efficacy.

## 2. Methods

We performed an economic evaluation from the US third-party payer perspective, following the recommendations of the Consolidated Health Economic Evaluation Reporting Standards (CHEERS) for economic evaluations [16].

In the base case scenario, it was assumed that all modeled patients were at the NAFL F0 health stage, which is the stage with the mildest symptoms of the disease. To ensure the robustness of our study, we conducted several calculations with the various initial disease stages of the cohort. This numerical experiment enabled us to assess the influence of starting conditions on the cohort’s behavior, particularly in cases where our model allowed for complete regression from NASH. The key endpoints in the clinical trials of NASH patients, as recommended by the US Food and Drugs Administration (FDA) [17], were NASH resolution and slowing down fibrosis progression. Therefore, the model utilized these endpoints to inform the regression from NASH to NAFL and the transition of fibrosis.

The analyses were conducted over a lifetime horizon, with a 1-year cycle length. This cycle length aligns effectively with the available data, and furthermore, the same cycle length has been employed in previously published models. Four different treatment strategies were considered, including two real ones and two theoretical ones. The first two strategies were lifestyle intervention and small molecule treatment, which are currently available in the market. The small molecule treatment was slowing down progression in early stages of fibrosis (F0–F2). The third strategy was a hypothetical biological treatment that aimed to prevent disease progression in the late stages of fibrosis (F3 and F4). The fourth strategy was a theoretical curative therapy that was assumed to cure the disease and return subjects from F4 stage to the initial stage of fibrosis (F0). 

A Markov cohort model was constructed, consisting of fourteen health states and one absorbing state that represented death:

- NAFL F0
- NAFL F1
- NAFL F2
- NASH F0
- NASH F1
- NASH F2
- F3 as advanced fibrosis
- 1st year with F4 as compensated cirrhosis
- Next year(s) with F4
- DC
- HCC
- Liver transplant after DC
- Liver transplant after HCC
- Post-liver transplant (PLT)

The model structure was inspired by the NASH model developed by the Institute for Clinical and Economic Review (ICER US) [13] and presented by Tapper et al. [18], which included comprehensive fibrosis and advanced complications-related health states defined according to the fibrosis stage. In addition, it included steatosis-specific health states defined according to the NAFLD activity score (NAS). 

**Figure 2:**
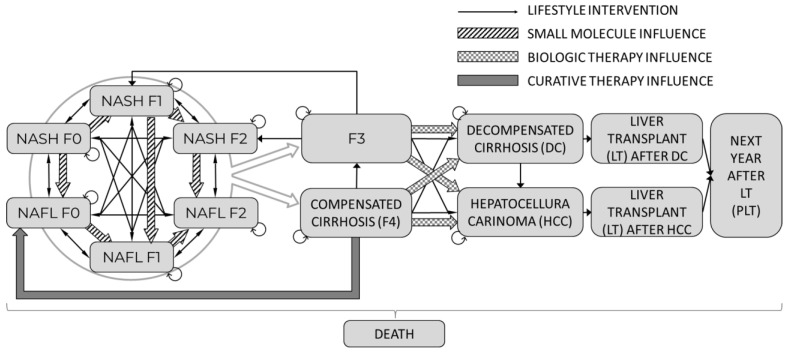
Flow chart of NAFL/NASH model.

The graphical structure of the model is presented in Figure 2. The cohort could progress and regress in the model between the stages of NAFL and NASH in different stages of fibrosis. Patients with NAFL or NASH and early stages of fibrosis (F0–F2) progressed to advanced fibrosis (F3) or compensated cirrhosis (F4). The latter fibrosis stages were not differentiated between NAFL and NASH as the severity of cirrhosis was considered to fully drive the associated outcomes. Patients with advanced fibrosis (F3) and compensated cirrhosis (CC) could transition to DC or HCC. The model’s mortality depends on the disease state, patient age, and sex. Furthermore, three types of mortality were allowed in the model:

- Cardiovascular death (CV death),
- Liver-related death,
- Other-cause mortality.

To illustrate all the potential influences of the explored treatment strategies, each transition marked with a thin black arrow represents the baseline lifestyle intervention pathway. Additionally, the impact of each particular treatment is shown by pattern-coded arrows that represent:

- Small molecule treatment with thick “zebra” line represents regressing towards NAFL from NASH into corresponding fibrosis stages and reducing fibrosis progression within NAFL or NASH.
- Biological therapy with thick checkered arrows shows a reduction of progression into DC and HCC from F3 and F4 states.
- Curative therapy with the thick grey arrow shows the transition probability into the initial stage of NAFL.

After treatment with curative therapy, subjects had a high probability of achieving full recovery and returning to the NAFL F0 stage. However, the possibility of relapse in these individuals was considered in the model.

Patients diagnosed with DC and HCC were considered eligible for LT in the model. It was assumed that individuals receiving LT would transition directly into the PLT health state, without accounting for potential disease relapse.

For each strategy, the following outcomes were evaluated: total life years (LY) and quality-adjusted life years (QALY); percentage of patients reaching advanced complications (DC, HCC, and LT); cause-specific mortality (liver-related mortality, fatal cardiovascular events (CVE), and other-cause mortality); and average cumulative costs (treatment acquisition costs, direct medical costs, and total costs). Then, the incremental cost-effectiveness ratio (ICER), expressed as total costs per QALY gained, was computed to compare the baseline and lifestyle intervention with other investigated types of treatments.

The general summary of model inputs is presented below. Additionally, all the values used in the model are available in Appendix A, Table A1.

## 3. Inputs

The model incorporated published data on the natural course of the disease, including probabilities sourced from Singh 2015 [19], Younossi et al., 2019 [12], and Tapper 2016 [18]. The costs and health-state utilities were based on the data provided by Younossi 2016. The efficacy of the treatments was informed by studies conducted by Tapper 2016 [18] and Zhang 2015 [20]. By utilizing these published data sources, the model aimed to provide a comprehensive and accurate representation of the disease and its potential treatment outcomes.

The baseline characteristics of the NAFL patients without advanced fibrosis were obtained from the NASH Clinical Research Network Study, which focused on patients with non-alcoholic fatty liver disease activity score (NAS) ≤ 4 [21]. The NAS score represented the sum of scores for steatosis, lobular inflammation, and ballooning, ranging from 0 to 8. Typically, subjects with a score equal to or above 4 were considered as having experienced NAFLD. The initial cohort was assumed to enter the model at an average age of 47.7, with females comprising 55.8% of the cohort

Transition probabilities across fibrosis stages originated from the study in which liver biopsies were conducted with a minimum one-year interval, reporting the annual fibrosis progression rates separately for patients with NAFL and NASH [19]. These transitions were recalculated using the same methodology as shown in the evidence report published by the ICER US [13]. The transition probability between NAFL and NASH was calculated based on Tapper, E.B. et al. [18]. Each transition from NAFL to NASH, or the opposite, was multiplied by the internal transition across fibrosis stages within NAFL/NASH. Transition probabilities to and across advanced complications and liver-related death were sourced from the evidence mentioned by the ICER US [13]. While developing the transition matrix, it was noticed that in the results from Singh, S. et al. [19], no NAFL subjects progressed from the F1 fibrosis stage into cirrhosis. However, there was one progression into cirrhosis from stage NAFL F0. Hence, the transition probability to cirrhosis from F0 was used also for F1 to mitigate this incoherence. 

The calculations regarding the efficacy of lifestyle intervention were made by taking into account weight loss among the patients who reached a certain weight reduction level [22]. For the small molecule treatment strategy, we assumed that it supported regression to NAFL from each corresponding NASH stage. Also, it reduced the risk of fibrosis progression in each stage of NASH and advanced fibrosis (F3). The biological therapy aimed to limit the progression from F3 and F4 into more severe stages of the disease. The curative therapy introduced the possibility of regressing into an early stage of NAFL from the F4 stage of the disease. Table 1 displays the breakdown of the key parameters’ values used in the model.

Liver-related and other-cause mortality were considered in the model together with the risk of fatal CVE based on the age- and sex-specific rates from the recent life tables [24] and cause-of-death data [25] published by the CDC. Liver-related mortality was adjusted in the model by incorporating a relative risk increase for patients with advanced cirrhosis (F3 or F4), DC, HCC, or after LT [13]. Additionally, to adjust the risk of fatal CVE, hazard ratios (HRs) were differentiated between stages F0 to F2, F3, and F4, based on a recent meta-analysis [26].

In this exploratory cost-effectiveness analysis, we investigate the potential for innovative therapy strategies similar to those presented by Binda et al. [27], showing a significant decrease in NAS score and fibrotic area. To test the impact on ICER, we investigated the efficacy of hypothetical therapies at different threshold values of 50%, 70%, and 90%. 

Utilities and costs for the model health states were retrieved from the Younossi et al. study [2], which used the micro-costing method to calculate costs and reported utilities elicited from Short Form-6D (SF-6D) in NAFLD patients. Age-adjusted utilities in the US population were taken from the evidence report published by the ICER US [13]. The costs related to lifestyle intervention and other treatment strategies were collected from the previously published cost-utility analyses [19,20]. Costs were adjusted to 2023 US dollars [28]. For the exploratory analysis, we assumed that the initial prices of biological and curative treatment were the same and equal to $500,000. The breakeven price of each therapy was calculated in the model considering different WTP thresholds of $50,000, $100,000, and $150,000 per QALY gained [29].

For deterministic sensitivity analyses (DSA), one-way sensitivity analyses were run by changing a single variable or assumption at a time. The DSA were conducted for all model parameters associated with uncertainty. Outcomes were computed using low and high model parameter values specified by confidence interval bounds when applicable (Table 1).

**Figure 3:**
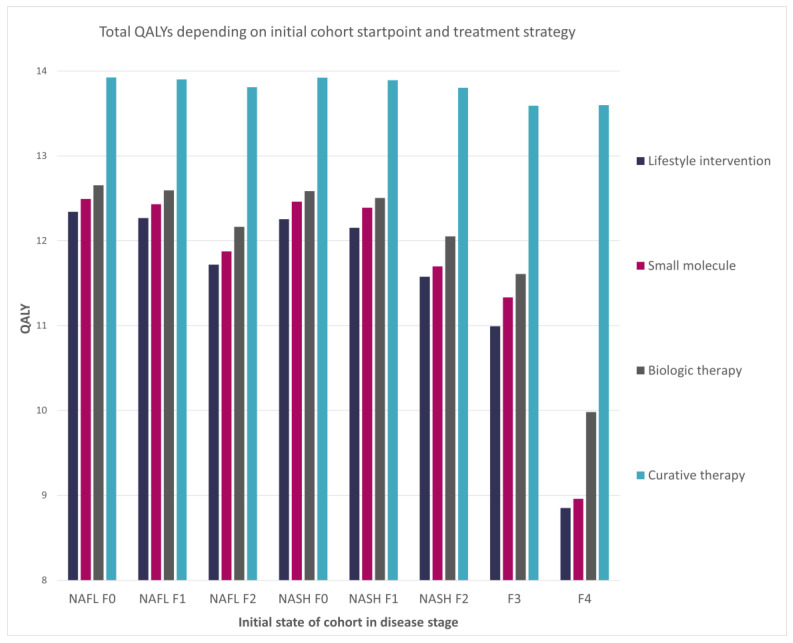
Impact of the cohort initial disease stage on the total QALYs per patient.

**Figure 4:**
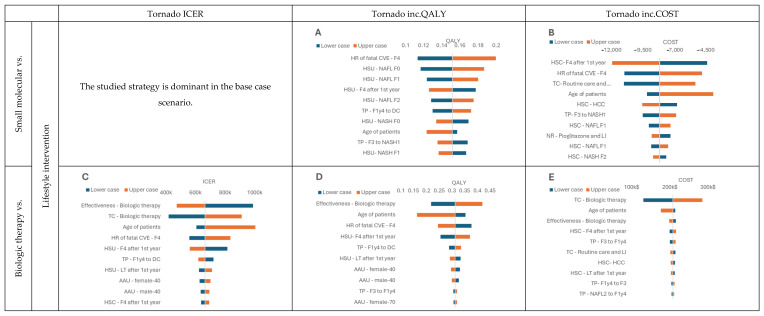

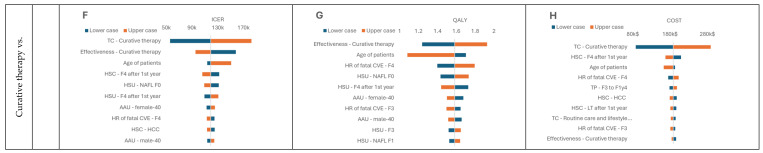
DSA results. (**A**) Tornado inc.QALY graph, Small molecular vs. Lifestyle intervention; (**B**) Tornado inc.COST graph, Small molecular vs. Lifestyle intervention; (**C**) Tornado ICER graph, Biologic therapy vs. Lifestyle intervention; (**D**) Tornado inc.QALY graph, Biologic therapy vs. Lifestyle intervention; (**E**) Tornado inc.COST graph, Biologic therapy vs. Lifestyle intervention; (**F**) Tornado ICER graph, Curative therapy vs. Lifestyle intervention; (**G**) Tornado inc.QALY graph, Curative therapy vs. Lifestyle intervention; (**H**) Tornado inc.COST graph Curative therapy vs. Lifestyle intervention Health state utility—HSU, Transition probabilities—TP, Health state costs—HSC, Treatment costs—TC, NASH resolution—NR, lifestyle intervention —LI, Age-adjusted utility—AAU.

**Figure 5:**
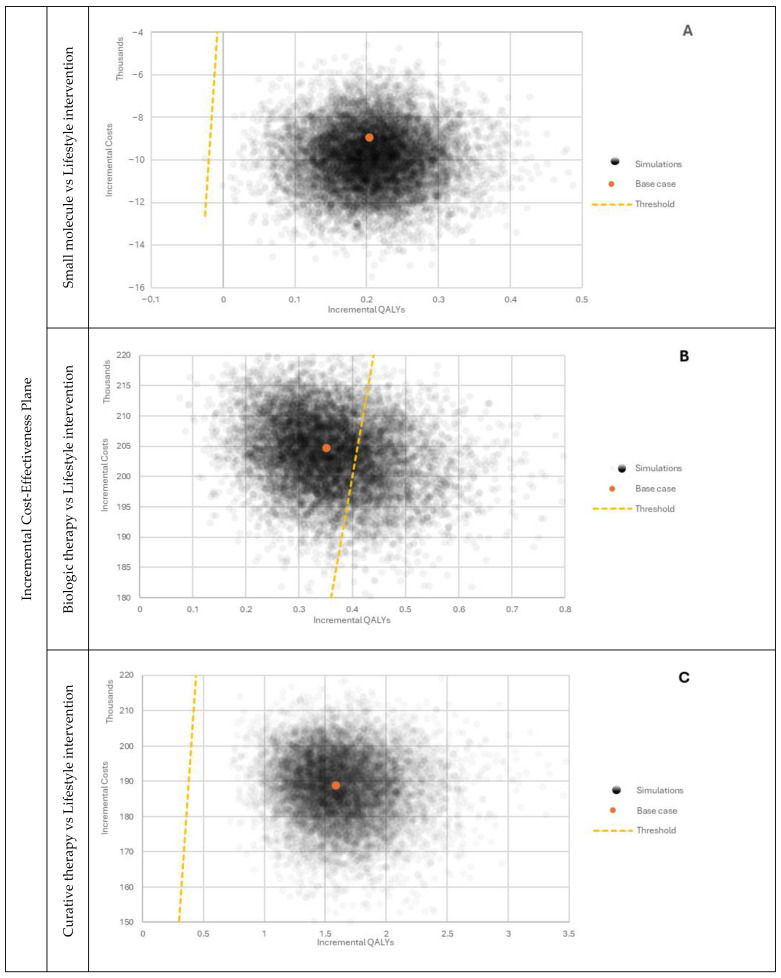
PSA outcomes comparing selected therapies with lifestyle intervention. (**A**) Incremental Cost-Effectiveness Plane, Small molecule vs. Lifestyle intervention; (**B**) Incremental Cost-Effectiveness Plane, Biologic therapy vs Lifestyle intervention; (**C**) Incremental Cost-Effectiveness Plane, Curative therapy vs. Lifestyle intervention.

**Figure A1:**
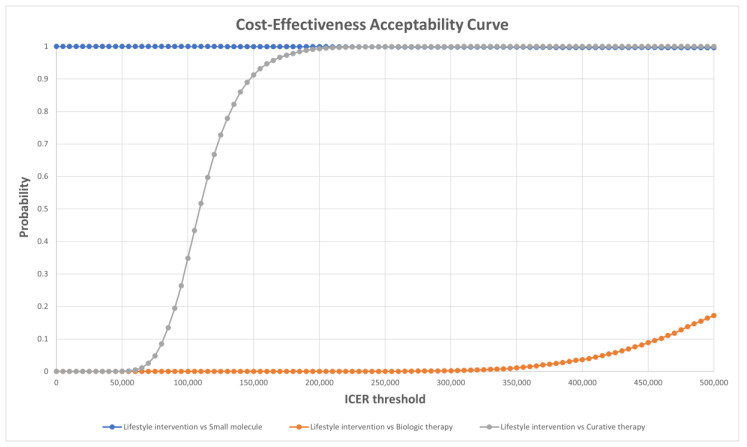
Cost-effectiveness acceptability curve of each treatment.

**Figure A2:**
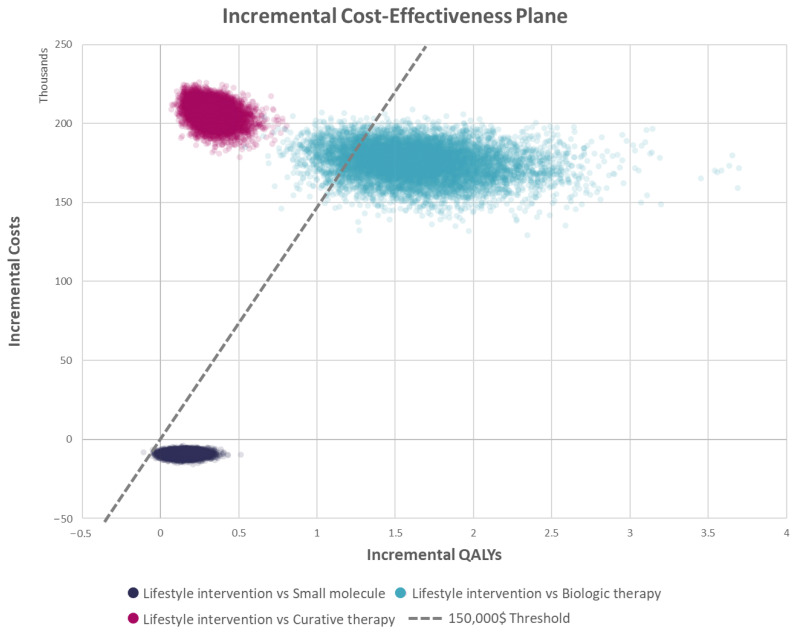
Incremental cost−effectiveness plane of each treatment.

For probabilistic sensitivity analysis (PSA), appropriate statistical distributions were assigned to input parameters. Values were drawn randomly from statistical distributions, including beta, gamma, Dirichlet, normal, and lognormal distributions. When it was not possible to obtain all the distribution parameters, a calibration based on the lower or higher bound of the DSA inputs was performed. Values were drawn from the prespecified distributions iteratively 10,000 times to generate distributions for ICERs. The results are presented graphically in (Figure 3, Figure 4 and Figure 5). The cost-effectiveness acceptability curves (CEAC) are available in the appendix (Figure A1), and the cost-effectiveness plane displaying each treatment in the shared plane is shown in (Figure A2).

## 4. Results

### 4.1. Cost-Effectiveness Results

The analysis performed showed that the hypothetical curative therapy provided the best results in terms of health outcomes. An extreme example of comparison showed that it was possible to achieve an additional increase of 1.58 QALYs for curative therapy compared with lifestyle intervention. The small molecule treatment yielded gains in QALYs and cost savings compared with lifestyle intervention, making it the dominant treatment. The results of the model’s base case scenarios are presented in Table 2.

The distribution of causes of death obtained in the model was similar between lifestyle intervention and small molecule therapy. However, in treatment with biological therapy, a specific decrease in liver-related deaths and an increase in deaths caused by fatal CVE were observed. The results of the curative therapy showed a significant reduction in deaths caused by the consequences of NASH/NAFL disease (Table 3).

The results shown in Table 4 indicate that all the outcomes behaved linearly depending on the efficacy. The reached values of incremental QALYs and costs highlight that the curative therapy, while holding comparable cost to the biological treatment, is reaching around five times higher incremental QALY gains. 

Table 5 shows the impact of the price of the therapy on the ICER. The price of a curative therapy could be more than twice the cost of a biological therapy. 

The resultant difference between strategies was highlighted in a comparison of lifetime QALYs depending on the initial state of the disease (Figure 3). The performance of the curative therapy was not substantially impacted by any analyzed initial stage of the disease. However, compared with the other treatments, it had the highest difference once implemented in the late F4 stage. This was most likely caused by the curative therapy initialization stage, which started from the F4 stage. 

### 4.2. Analysis of Key Drivers of Cost-Effectiveness (DSA–PSA)

While performing deterministic sensitivity analysis, we analyzed the influence of input parameters on model outcomes such as ICER, QALYs, and costs. Due to the dominance in the base case, we could not generate the tornado plot for the ICER variable for comparison of lifestyle intervention with small molecule therapy (Figure 4A,B). However, we observed for this comparison that utility and costs of particular health states had the most significant impact on the model results. When analyzing the comparison with biological therapy, we observed that one of the most influential factors was its assumed effectiveness (Figure 4C–E). The curative therapy behaved similarly to the small molecule therapy (Figure 4F–H) regarding the influence of different parameters. However, it is worth pointing out that modification of the curative therapy effectiveness and utility of patients with NAFL F0 substantially influenced QALY gained. This outcome was expected because those are the dimensions that curative therapy depends on.

The performed probabilistic sensitivity analysis showed how model outcomes are sensitive to parameter changes within the assumed distributions. As observed when comparing the small molecule with lifestyle intervention, most results were located in the fourth quarter of the CE plane (Figure 5A). The majority of the simulation outputs fell below the willingness-to-pay threshold. In the analysis of the biological therapy, all of the simulation results were within the first quarter of the graph (Figure 5B). However, there was a noticeable split between outputs divided by the willingness-to-pay threshold line. The output from comparing the curative therapy with lifestyle intervention was also located in the first quarter of the plot (Figure 5C) with all the simulation outcomes below the willingness-to-pay threshold. 

## 5. Discussion

The study revealed that the curative treatment involving cell-based therapy, with the assumed efficacy of 70% of patients cured and assumed price of $500,000, was a cost-effective treatment compared with the other strategies. Although it incurred higher costs, it also generated higher QALY gains, resulting in an ICER below the commonly accepted willingness-to-pay threshold of $150,000 per QALY in the US. This suggested that curative therapy could be viable for patients with NAFL or NASH, especially those at higher risk of disease progression. The study highlighted the potential significance of therapies aimed at curing rather than merely halting disease progression. Nonetheless, each of the analyzed therapies with the assumed efficacy could be cost-effective compared with lifestyle intervention at a relatively high price. 

The sensitivity analyses showed that the results were robust to variations in model parameters. Finally, to check the performance of our model, we compared the results of lifestyle intervention from our model with the initial data used in other reviewed models [13,14]. After adopting input values to be the same as those in the compared models (while including a similar model structure), we obtained results for comparison. The incremental QALYs between small molecule and lifestyle interventions were similar between our model and the ICER US model (0.70 vs. 0.61). It is worth noting that small differences in reaching particular QALY values could have occurred because the compared models were not the same but shared similarities in a core structure. In particular our model did not include the cardiovascular events. Nevertheless, we concluded that, despite the small disparity, the results obtained confirm the reliability of our model.

Moreover, our model can be considered conservative due to the further inclusion of health states corresponding to NAFL, which were not considered by ICER US. Once we had introduced the extension of the NAFL and NASH stages, we observed that lifestyle intervention kept subjects in less advanced states for longer periods, which affected the QALY results. When considering the possibility of regression towards NAFL in the early fibrosis stages, our model estimated a much lower difference in QALYs between small molecule and lifestyle interventions (0.20) than the model developed by ICER US (0.61).

However, we acknowledge some limitations of the study. One limitation was that the model did not consider the potential side effects and long-term safety of the treatments, especially for biological and curative therapy. Additionally, the efficacy and costs of some treatments were based on limited evidence, which could have influenced the results. Moreover, the model only considered a US healthcare system perspective, and the results may not be generalizable to other healthcare systems. Lastly, the lifestyle intervention therapy primarily focused on subjects’ weight loss and did not encompass the entire spectrum of parameters that could be addressed in NAFL or NASH. We acknowledge the simplification of subject behavioral change; however, such an approach is considered standard in the cost-effectiveness modeling for NASH. 

This finding could have important implications, highlighting the potential benefits of innovative therapies for managing NAFL and NASH and their cost-effectiveness. However, further research is needed to validate the efficacy and safety of such therapies and evaluate their long-term outcomes.

The presented methodology had novelty in extending the model population by including NAFL-diseased subjects. Thus, a broader spectrum of potential patients was included in the evaluation compared with previous studies. In addition, the previous version of the model had a different approach to calculating transitions within the early stages of NASH progression. Despite initial differences, the model structure was able to reach similar outcomes to the model presented by ICER US, and can be considered as its extension through the inclusion of NAFL health states and consideration of new therapies. 

As the treatments evaluated in the model are theoretical, certain assumptions have been made concerning the fixed treatment cost within the model. This limitation is inherent in the study, and one of its consequences pertains to the interpretation of PSA results. In practice, some treatment outcomes, such as life extension, may be associated with an increase in treatment cost, a factor not currently observable. This limitation is expected to be addressed when more information on the posology of potential treatments becomes available. 

Upon reviewing the literature, we concluded that assuming the direct results of Singh, S. et al. [19] as a distribution source held a certain level of error due to the limited number of observations and cases enlisted in the study. Thus, we considered the data as a foundation for further development. Furthermore, as we reviewed models available in the literature, the methodology presented by ICER US was deemed to have the most appropriate approach for mitigating limited access to data on disease progression. Therefore, we adapted the methodology proposed by ICER US in our calculations. 

The other opinion about ICER US methodology was found while reviewing Javanbakht, M. et al. [14]. The authors pointed out that, despite such an approach being a valid option, it considered at least one step progression of the fibrosis stage at a time. The origin of such interpretation might have had a source in the multiplication weight value that was applied in the calculation procedure. The wage to calculate the transition probabilities was taken from Younossi, Z.M. et al. [12], representing the transition probability at least in one step of fibrosis. The wage values for improvement and worsening varied. On one hand, it was possible to claim that there was uncertainty regarding the assumption that the potential distribution of accelerated transition was unknown if we used such a wage. On the other hand, the data distillation procedure to obtain the progression proportion included numerous studies and participants that surpassed, in terms of amount, the analyzed participants in Singh, S. et al. [19]. Thus, it significantly reduced the coincidence of group proportion in analyzing patients, for example, the lack of connection in the Singh, S. et al. [19] NAFL matrix between F1 to F4, although there was a connection between F0 and F4. Due to the highly specific studies, access to the data was limited, highly anticipated, and usually challenging to obtain. Thus, formulating models that operate on a population level necessitated specific generalization layers in calculations.

It has to be noted that, due to the exploratory nature of this analysis, we did not consider potential side effects or safety issues related to new therapies. Their inclusion would require more precise data, which are not available. The inclusion of any side effects in the model would affect tested treatments by reducing incremental gains of QALYs, increasing the total cost of the investigated treatment. Consequently, it might impact the overall cost-effectiveness.

In the current version of our model, we did not include additional cardiovascular events triggered by the accumulation of pharmacological compounds due to their dosage, as shown in ICER US [13] and Javanbakht, M. et al. [14]. It should also be noted that this model version was a simplification of the actual clinical scenario, as it was shown in a publication on post-liver transplantation outcomes by Anstee, Q. M. et al. [30] that patients who underwent LT had a significant risk of NASH/NAFL re-emergence due to the difficulty in evaluating the health status of the donor’s liver. Therefore, such events were not included in the model for this study but were considered in future development steps. 

It is important to note that we assumed that published results omitted any misinterpretation, which could have been a potential source of inaccuracy. As shown by Anstee Q.M et al. [31], there is a space for biased judgment while evaluating fibrosis stage classification. Depending on a specialization background, the practitioner could have had a slightly different interpretation of the stage of disease progression. Such a phenomenon was not considered in the current stage of model development. However, the impact of the initial stage of the disease on model results was widely explored.

Furthermore, the current state of the art of our model included theoretical treatments and their outcomes. In contrast, several potential treatments for NAFL and NASH have been under development for a couple of years. Extending the modeling exercise to account for these therapies could have been a field for additional research.

## Figures and Tables

**Table 1:** Summary of model inputs.

Model Parameter	Base Case	Distribution	Low Value	High Value	Source
**General settings**					
Discount rate for costs	3.00%	Normal	1.50%	5.00%	Weinstein 1996 [23]
Discount rate for outcomes	3.00%	Normal	1.50%	5.00%	
Percentage of female	56%	Beta	50%	61%	Brunt 2011 [21]
Age of patients	47.70	Normal	45.90	56.10	
**Costs**					
Treatment costs					
Treatment costs—Curative therapy	$500,000.00	Lognormal	$350,000.00	$650,000.00	Assumed
Treatment costs—Routine care and pioglitazone	$2311.00	Lognormal	$1617.96	$3004.79	Tapper 2016 [18]
Treatment costs—Routine care and lifestyle intervention	$2083.00	Lognormal	$1458.43	$2708.51	Zhang 2015 [20]
Treatment costs—Biologic therapy	$500,000.00	Lognormal	$350,000.00	$650,000.00	Assumed
Health state costs, annual					
Health state costs—NAFL F0	$2882.60	Gamma	$2017.82	$3747.38	Younossi 2016 [3]
Health state costs—NAFL F1	$5765.20	Gamma	$4035.64	$7494.76	
Health state costs—NAFL F2	$8647.80	Gamma	$6053.46	$11,242.14	
Health state costs—NASH F0	$4118.00	Gamma	$2882.60	$5353.40	
Health state costs—NASH F1	$8236.00	Gamma	$5765.20	$10,706.80	
Health state costs—NASH F2	$12,354.00	Gamma	$8647.80	$16,060.20	
Health state costs—F3	$17,904.74	Gamma	$12,533.32	$23,276.16	
Health state costs—F4	$29,688.12	Gamma	$20,781.68	$38,594.56	
Health state costs—DC	$106,370.53	Gamma	$74,459.37	$138,281.69	
Health state costs—HCC	$215,504.24	Gamma	$150,852.97	$280,155.51	
Health state costs—LT 1st year	$215,504.24	Gamma	$150,852.97	$280,155.51	
Health state costs—LT after 1st year	$53,043.06	Gamma	$37,130.14	$68,955.98	
Transition probabilities, annual					
Transition probabilities	Singh 2015 [19], Younossi et al., 2019 [12], Tapper 2016 [18], Zhang 2015 [20], ICER NASH Draft Report 2023 [13]				
Utilities, annual					
Health state utility—NAFL, NASH F0–F2	0.76	Beta	0.68	0.84	Younossi 2016 [3]
Health state utility—F3	0.73	Beta	0.66	0.80	
Health state utility—F4	0.66	Beta	0.59	0.73	
Health state utility—DC	0.57	Beta	0.51	0.63	
Health state utility—HCC	0.50	Beta	0.45	0.55	
Health state utility—LT	0.73	Beta	0.66	0.80	
Efficacy, annual					
NASH resolution					
NASH resolution—Lifestyle intervention—Weightloss < 5%	10.24%	Beta	8.20%	12.29%	Tapper 2016 [18]
NASH resolution—Lifestyle intervention—Weightloss 5–10%	42.37%	Beta	33.90%	50.85%	
NASH resolution—Lifestyle intervention—Weightloss > 10%	89.65%	Beta	71.72%	107.59%	
NASH resolution—Pioglitazone and lifestyle intervention	52.7%.	Beta	42.16%	63.24%	Zhang 2015 [20]
Fibrosis progression					
Fibrosis progression—Lifestyle intervention—Weightloss < 5%	100.00%	Beta	80.00%	120.00%	Tapper 2016 [18]
Fibrosis progression—Lifestyle intervention—Weightloss 5–10%	98.93%	Beta	79.15%	118.72%	
Fibrosis progression—Lifestyle intervention—Weightloss > 10%	40.81%	Beta	32.65%	48.98%	
Fibrosis progression—Small molecular	40.81%	Beta	32.65%	48.98%	
Probability of reversing cirrhosis/avoiding further progression					
Probability of avoiding further progression—Biologic therapy	70.00%	Beta	56.00%	84.00%	Assumed
Probability of reversing cirrhosis—Curative therapy	70.00%	Beta	56.00%	84.00%	
Weight loss in patients with lifestyle intervention					
Percentage—Weightloss < 5%	80.19%	Beta	64.16%	96.23%	Tapper 2016 [18]
Percentage—Weightloss 5–10%	12.87%	Beta	10.30%	15.45%	
Percentage—Weightloss > 10%	6.92%	Beta	5.54%	8.31%	

**Table 2:** Base case results of NAFL/NASH cohort model.

	Lifestyle Intervention		Small Molecule		Biologic Therapy		Curative Therapy	
	Undiscounted	Discounted	Undiscounted	Discounted	Undiscounted	Discounted	Undiscounted	Discounted
**Health outcomes**								
**Total LYs**	26.62	17.64	27.20	17.88	27.70	18.15	31.34	19.59
**Total QALYs**	18.33	12.341	18.79	12.54	18.98	12.65	21.96	13.93
**Cost outcomes**								
**Direct medical costs**	$511,617	$302,495	$482,053	$284,990	$467,135	$274,729	$298,730	$179,431
**Treatment costs**	$31,226	$22,378	$45,246	$30,953	$399,587	$258,303	$560,547	$334,262
**Total costs**	$542,843	$324,873	$527,299	$315,943	$866,722	$533,032	$859,277	$513,693

**Table 3:** Distribution of events and causes of deaths observed in the model.

	Lifestyle Intervention	Small Molecule	Biologic Therapy	Curative Therapy
**Percentage of patients experiencing**				
**DC**	42.0%	37.8%	21.4%	4.1%
**HCC**	31.8%	28.7%	15.2%	3.4%
**LT**	1.9%	1.7%	0.9%	0.2%
**Mortality**				
**Liver-related**	19.4%	17.5%	9.7%	2.6%
**Fatal CVE**	47.4%	47.1%	54.3%	45.3%
**Other cause**	34.2%	36.6%	37.0%	54.6%

**Table 4:** Incremental discounted results for different levels of efficacy.

Outcome	Assumed Efficacy	Biologic Therapy	Curative Therapy	Biologic Therapy	Curative Therapy
		vs. Lifestyle		vs. Small Molecule	
**Incr. QALY**	50%	0.23	1.11	0.03	0.91
	70%	0.35	1.58	0.15	1.38
	90%	0.49	2.07	0.28	1.86
**Incr. Costs**	50%	$214,674	$198,613	$223,605	$207,544
	70%	$204,680	$188,771	$213,611	$197,702
	90%	$192,407	$183,910	$201,338	$192,841

**Table 5:** Economically justifiable price at different levels of WTP and treatment efficacy.

WTP	Assumed Efficacy	Biologic Therapy	Curative Therapy	Biologic Therapy	Curative Therapy
		vs. Lifestyle		vs. Small Molecule	
**50,000**	50%	$105,666	$268,127	$68,581	$237,207
	70%	$137,785	$336,138	$100,837	$307,586
	90%	$175,757	$388,931	$138,947	$362,611
**100,000**	50%	$128,429	$357,984	$71,609	$310,612
	70%	$171,773	$454,646	$115,164	$410,901
	90%	$222,570	$531,460	$166,173	$491,135
**150,000**	50%	$151,191	$447,841	$74,638	$384,016
	70%	$205,761	$573,153	$129,492	$514,216
	90%	$269,382	$673,989	$193,398	$619,658

## Data Availability

The original contributions presented in the study are included in the article, further inquiries can be directed to the corresponding author/s.

## List of Abbreviations

NAFLD	Non-alcoholic fatty liver disease
NAFL	non-alcoholic fatty liver
NASH	non-alcoholic steatohepatitis
F0	initial stage of fibrosis progression in NAFL or NASH, considered as a healthy person
F1	first stage of fibrosis progression in NAFL or NASH
F2	second stage of fibrosis progression in NAFL or NASH
F3	third stage of fibrosis progression
F4	fourth stage of fibrosis progression or compensated cirrhosis
DC	Decompensated Cirrhosis
HCC	Hepatocellular Carcinoma
LT	Liver transplant
PLT	Post-liver transplant
CHEERS	Consolidated Health Economic Evaluation Reporting Standards
LY	life years
ICER	incremental cost-effectiveness ratio
CVE	fatal cardiovascular events
QALY	quality-adjusted life-years
DSA	deterministic sensitivity analyses
PSA	probabilistic sensitivity analysis
NAS	NAFLD activity score
CC	compensated cirrhosis
SF-6D	Short Form-6D
CEAC	cost-effectiveness acceptability curve
HR	hazard ratio
ICER US	Institute for Clinical and Economic Review
FDA	US Food and Drugs Administration

## Appendix A

**Table A1:** Summary of all Input values.

Parameter	Base Case	Distribution	Low Value	High Value	Source
**General settings**					
Discount rate for costs	3.00%	Normal	1.50%	5.00%	Weinstein 1996 [23]
Discount rate for outcomes	3.00%	Normal	1.50%	5.00%	Weinstein 1996 [23]
Percentage of female	56%	Beta	50%	61%	Brunt 2011 [21]
Age of patients	47.70	Normal	45.90	56.10	Brunt 2011 [21]
**Costs**					
**Treatment costs**					
Treatment costs—Curative therapy	$500,000	Lognormal	$350,000	$650,000	Assumed
Treatment costs—Routine care and pioglitazone	$2311.38	Lognormal	$1617.96	$3004.79	Tapper 2016 [18]
Treatment costs—Routine care and lifestyle intervention	$2083.47	Lognormal	$1458.43	$2708.51	Zhang 2015 [20]
Treatment costs—Biologic therapy	$500,000	Lognormal	$350,000	$650,000	Assumed
**Health state costs**					
Health state costs—NAFL F0	$2882.60	Gamma	$2017.82	$3747.38	Younossi 2016 [3]
Health state costs—NAFL F1	$5765.20	Gamma	$4035.64	$7494.76	
Health state costs—NAFL F2	$8647.80	Gamma	$6053.46	$11,242.14	
Health state costs—NASH F0	$4118.00	Gamma	$2882.60	$5353.40	
Health state costs—NASH F1	$8236.00	Gamma	$5765.20	$10,706.80	
Health state costs—NASH F2	$12,354.00	Gamma	$8647.80	$16,060.20	
Health state costs—F3	$17,904.74	Gamma	$12,533.32	$23,276.16	
Health state costs—F4 1st year	$29,688.12	Gamma	$20,781.68	$38,594.56	
Health state costs—F4 after 1st year	$29,688.12	Gamma	$20,781.68	$38,594.56	
Health state costs—DC	$106,370.53	Gamma	$74,459.37	$138,281.69	
Health state costs—HCC	$215,504.24	Gamma	$150,852.97	$280,155.51	
Health state costs—LT 1st year after DC	$215,504.24	Gamma	$150,852.97	$280,155.51	
Health state costs—LT 1st year after HCC	$215,504.24	Gamma	$150,852.97	$280,155.51	
Health state costs—LT after 1st year	$53,043.06	Gamma	$37,130.14	$68,955.98	
**Transition probabilities & HRs**					
**Transition probabilities**					
Transition probabilities—NAFL0 to NAFL1	14.66%	Dirichlet	13.19%	16.12%	Singh 2015 [19], Younossi et al., 2019 [12]
Transition probabilities—NAFL0 to NAFL2	7.33%	Dirichlet	6.60%	8.06%	
Transition probabilities—NAFL0 to NASH0	1.98%	Dirichlet	1.79%	2.18%	Singh 2015 [19], Younossi et al., 2019 [12], Tapper 2016 [18]
Transition probabilities—NAFL0 to NASH1	0.47%	Dirichlet	0.43%	0.52%	
Transition probabilities—NAFL0 to NASH2	0.14%	Dirichlet	0.12%	0.15%	
Transition probabilities—NAFL0 to F3	3.66%	Dirichlet	3.30%	4.03%	Singh 2015 [19], Younossi et al., 2019 [12]
Transition probabilities—NAFL0 to F1y4	0.92%	Dirichlet	0.82%	1.01%	
Transition probabilities—NAFL1 to F3	6.14%	Dirichlet	5.53%	6.76%	
Transition probabilities—NAFL1 to F1y4	0.00%	Dirichlet	0.00%	0.00%	
Transition probabilities—NAFL2 to F3	13.59%	Dirichlet	12.23%	14.95%	
Transition probabilities—NAFL2 to F1y4	6.79%	Dirichlet	6.11%	7.47%	
Transition probabilities—NAFL1 to NAFL0	22.42%	Dirichlet	20.18%	24.67%	
Transition probabilities—NAFL1 to NAFL2	14.33%	Dirichlet	12.90%	15.77%	
Transition probabilities—NAFL1 to NASH0	0.63%	Dirichlet	0.57%	0.69%	Singh 2015 [19], Younossi et al., 2019 [12], Tapper 2016 [18]
Transition probabilities—NAFL1 to NASH1	1.53%	Dirichlet	1.38%	1.68%	
Transition probabilities—NAFL1 to NASH2	0.34%	Dirichlet	0.31%	0.38%	
Transition probabilities—NAFL2 to NAFL0	9.02%	Dirichlet	8.12%	9.92%	Singh 2015 [19], Younossi et al., 2019 [12]
Transition probabilities—NAFL2 to NAFL1	13.53%	Dirichlet	12.18%	14.88%	
Transition probabilities—NAFL2 to NASH0	0.15%	Dirichlet	0.13%	0.16%	Singh 2015 [19], Younossi et al., 2019 [12], Tapper 2016 [18]
Transition probabilities—NAFL2 to NASH1	0.49%	Dirichlet	0.44%	0.53%	
Transition probabilities—NAFL2 to NASH2	1.54%	Dirichlet	1.38%	1.69%	
Transition probabilities—NASH0 to NAFL0	19.88%	Dirichlet	17.89%	21.87%	
Transition probabilities—NASH0 to NAFL1	0.55%	Dirichlet	0.50%	0.61%	
Transition probabilities—NASH0 to NAFL2	0.28%	Dirichlet	0.25%	0.30%	
Transition probabilities—NASH0 to NASH1	16.75%	Dirichlet	15.07%	18.42%	Singh 2015 [19], Younossi et al., 2019 [12]
Transition probabilities—NASH0 to NASH2	4.79%	Dirichlet	4.31%	5.26%	
Transition probabilities—NASH0 to F3	2.39%	Dirichlet	2.15%	2.63%	
Transition probabilities—NASH0 to F1y4	2.39%	Dirichlet	2.15%	2.63%	
Transition probabilities—NASH1 to F3	6.76%	Dirichlet	6.08%	7.44%	
Transition probabilities—NASH1 to F1y4	1.35%	Dirichlet	1.22%	1.49%	
Transition probabilities—NASH2 to F3	10.19%	Dirichlet	9.17%	11.21%	
Transition probabilities—NASH2 to F1y4	10.19%	Dirichlet	9.17%	11.21%	
Transition probabilities—NASH1 to NAFL0	0.84%	Dirichlet	0.76%	0.93%	Singh 2015 [19], Younossi et al., 2019 [12], Tapper 2016 [18]
Transition probabilities—NASH1 to NAFL1	19.88%	Dirichlet	17.89%	21.87%	
Transition probabilities—NASH1 to NAFL2	0.54%	Dirichlet	0.49%	0.59%	
Transition probabilities—NASH1 to NASH0	22.21%	Dirichlet	19.99%	24.43%	Singh 2015 [19], Younossi et al., 2019 [12]
Transition probabilities—NASH1 to NASH2	12.17%	Dirichlet	10.95%	13.38%	
Transition probabilities—NASH2 to NAFL0	0.34%	Dirichlet	0.31%	0.37%	Singh 2015 [19], Younossi et al., 2019, Tapper 2016 [18]
Transition probabilities—NASH2 to NAFL1	0.51%	Dirichlet	0.46%	0.56%	
Transition probabilities—NASH2 to NAFL2	19.88%	Dirichlet	17.89%	21.87%	
Transition probabilities—NASH2 to NASH0	5.15%	Dirichlet	4.64%	5.67%	Singh 2015 [19], Younossi et al., 2019 [12]
Transition probabilities—NASH2 to NASH1	17.18%	Dirichlet	15.46%	18.89%	
Transition probabilities—F3 to NASH1	5.62%	Dirichlet	5.06%	6.18%	
Transition probabilities—F3 to NASH2	5.62%	Dirichlet	5.06%	6.18%	
Transition probabilities—F3 to F1y4	10.26%	Dirichlet	9.24%	11.29%	
Transition probabilities—F3 to DC	0.32%	Dirichlet	0.29%	0.35%	ICER NASH Draft Report 2023 [13]
Transition probabilities—F3 to HCC	0.24%	Dirichlet	0.22%	0.26%	
Transition probabilities—NASH_B to NAFL1	52.70%	Dirichlet	47.43%	57.97%	Zhang 2015 [20]
Transition probabilities—NASH_B0 to NASH_B1	13.23%	Dirichlet	11.91%	14.55%	
Transition probabilities—NASH_B0 to NASH_B2	3.78%	Dirichlet	3.40%	4.16%	
Transition probabilities—NASH_B1 to NASH_B2	9.61%	Dirichlet	8.65%	10.57%	
Transition probabilities—NASH_B0 to F3	1.89%	Dirichlet	1.70%	2.08%	
Transition probabilities—NASH_B1 to F3	5.34%	Dirichlet	4.81%	5.87%	
Transition probabilities—NASH_B2 to F3	8.05%	Dirichlet	7.25%	8.86%	
Transition probabilities—F1y4 to F3	27.27%	Dirichlet	24.55%	30.00%	Singh 2015 [19], Younossi et al., 2019 [12]
Transition probabilities—F1y4 to DC	4.22%	Dirichlet	3.79%	4.64%	ICER NASH Draft Report 2023 [13]
Transition probabilities—F1y4 to HCC	2.11%	Dirichlet	1.90%	2.32%	
Transition probabilities—F_B3 to F1y4	4.80%	Dirichlet	4.32%	5.28%	Zhang 2015 [20]
Transition probabilities—DC to HCC	3.45%	Dirichlet	3.11%	3.80%	ICER NASH Draft Report 2023 [13]
Transition probabilities—DC to LT	37.45%	Dirichlet	33.71%	41.20%	
Transition probabilities—HCC to LT	35.20%	Dirichlet	31.68%	38.72%	
**Hazard ratios**					
HR of fatal CVE—NAFL F0	1.00	Normal	0.65	1.16	Hagström, H. et. al. [26]
HR of fatal CVE—NAFL F1	1.01	Normal	0.70	1.46	
HR of fatal CVE—NAFL F2	1.60	Normal	1.09	2.39	
HR of fatal CVE—NASH F0	1.00	Normal	0.65	1.16	
HR of fatal CVE—NASH F1	1.01	Normal	0.61	1.36	
HR of fatal CVE—NASH F2	1.85	Normal	0.70	1.76	
HR of fatal CVE—F3	3.04	Normal	1.94	4.78	
HR of fatal CVE—F4	6.53	Normal	3.55	12.03	
**Utilities**					
**Health state utility**					
Health state utility—NAFL F0	0.76	Beta	0.68	0.84	Younossi 2016 [3]
Health state utility—NAFL F1	0.76	Beta	0.68	0.84	
Health state utility—NAFL F2	0.76	Beta	0.68	0.84	
Health state utility—NASH F0	0.76	Beta	0.68	0.84	
Health state utility—NASH F1	0.76	Beta	0.68	0.84	
Health state utility—NASH F2	0.76	Beta	0.68	0.84	
Health state utility—F3	0.73	Beta	0.66	0.80	
Health state utility—F4 1st year	0.66	Beta	0.59	0.73	
Health state utility—F4 after 1st year	0.66	Beta	0.59	0.73	
Health state utility—DC	0.57	Beta	0.51	0.63	
Health state utility—HCC	0.50	Beta	0.45	0.55	
Health state utility—LT 1st year after DC	0.73	Beta	0.66	0.80	
Health state utility—LT 1st year after HCC	0.73	Beta	0.66	0.80	
Health state utility—LT after 1st year	0.73	Beta	0.66	0.80	
**Age-adjusted utilities**					
Age-adjusted utility—male-40	0.89	Beta	0.80	0.98	Tapper 2016 [18]
Age-adjusted utility—male-50	0.86	Beta	0.78	0.95	
Age-adjusted utility—male-60	0.84	Beta	0.76	0.92	
Age-adjusted utility—male-70	0.80	Beta	0.72	0.88	
Age-adjusted utility—male-80	0.78	Beta	0.70	0.86	
Age-adjusted utility—female-40	0.86	Beta	0.78	0.95	
Age-adjusted utility—female-50	0.84	Beta	0.75	0.92	
Age-adjusted utility—female-60	0.81	Beta	0.73	0.89	
Age-adjusted utility—female-70	0.77	Beta	0.69	0.85	
Age-adjusted utility—female-80	0.72	Beta	0.65	0.80	
**Efficacy**					
**NASH resolution**					
NASH resolution—Lifestyle intervention—Weightloss < 5%	10.24%	Beta	8.20%	12.29%	Tapper 2016 [18]
NASH resolution—Lifestyle intervention—Weightloss 5–10%	42.37%	Beta	33.90%	50.85%	
NASH resolution—Lifestyle intervention—Weightloss > 10%	89.66%	Beta	71.72%	107.59%	
NASH resolution—Pioglitazone and lifestyle intervention	52.70%	Beta	42.16%	63.24%	Zhang 2015 [20]
**Fibrosis progression**					
Fibrosis progression—Lifestyle intervention—Weightloss < 5%	100.00%	Beta	80.00%	120.00%	Tapper 2016 [18]
Fibrosis progression—Lifestyle intervention—Weightloss 5–10%	98.93%	Beta	79.15%	118.72%	
Fibrosis progression—Lifestyle intervention—Weightloss > 10%	40.81%	Beta	32.65%	48.98%	
Fibrosis progression—Small molecular	40.81%	Beta	32.65%	48.98%	
**Probability of reversing cirrhosis/avoiding further progression**					
Effectiveness—Biologic therapy	70.00%	Beta	56.00%	84.00%	Assumed
Effectiveness—Curative therapy	70.00%	Beta	56.00%	84.00%	Assumed
**Weight loss in patients with lifestyle intervention**					
Percentage—Weightloss < 5%	80.19%	Beta	64.16%	96.23%	Tapper 2016 [18]
Percentage—Weightloss 5–10%	12.88%	Beta	10.30%	15.45%	
Percentage—Weightloss > 10%	6.93%	Beta	5.54%	8.31%	
